# The gut-brain axis mediates precocious puberty induced by environmentally relevant low-dose endocrine-disrupting chemical mixtures

**DOI:** 10.3389/fendo.2025.1728811

**Published:** 2026-01-22

**Authors:** Hui Wu, Guo Wei, Shuo Huang, Lingyu Wan, Qin Xu, Congfu Huang

**Affiliations:** 1 Child Healthcare Department, PanYu District Maternal and Child Health Care Hospital, Guangzhou (PanYu District He Xian Memorial Hospital), Guangzhou, China; 2 Child Healthcare Department, Longgang District Maternity and Child Healthcare Hospital of Shenzhen City [Affiliated Shenzhen Women and Children’s Hospital (Longgang)] of Shantou University Medical College, Shenzhen, China; 3 Department of Pediatrics, Longgang District Maternity and Child Healthcare Hospital of Shenzhen City [Affiliated Shenzhen Women and Children’s Hospital (Longgang)] of Shantou University Medical College, Shenzhen, China

**Keywords:** chemical mixtures, endocrine-disrupting chemicals, gut-brain axis, low-dose exposure, microbiome, precocious puberty, systematic review

## Abstract

**Background:**

The global rise in precocious puberty (PP) is increasingly linked to exposure to endocrine-disrupting chemicals (EDCs). However, the mechanisms by which environmentally relevant, low-dose mixtures of EDCs influence PP remain inadequately explained by direct endocrine disruption.

**Objective:**

This systematic review evaluates a novel hypothesis: that disruption of the gut-brain axis (GBA) serves as a pivotal mechanism in EDC mixture-induced PP.

**Methods:**

We synthesized evidence from 87 studies (45 human, 32 animal, 10 *in vitro*) following PRISMA 2020 guidelines. An exploratory Random Forest analysis was employed to identify key mediators and estimate the relative contribution of the GBA pathway.

**Results:**

Perinatal exposure to low-dose EDC mixtures consistently induced gut dysbiosis, characterized by reduced microbial diversity (Shannon Δ = −1.8), a 40% decrease in Lactobacillus, and a 1.5-fold increase in Bacteroides. This dysbiosis was linked to impaired production of butyrate (↓50%) and secondary bile acids, increased intestinal permeability (FITC-dextran ↑80%), and systemic inflammation (IL-6 ↑1.8-fold). Fecal microbiota transplantation from PP donors into germ-free mice recapitulated early pubertal onset, supporting a causal role for gut microbiota. Exploratory modeling suggested that mediators within the GBA pathway could be associated with a large share (approximately 68%) of the model-internal variance explanation for PP risk at low experimental doses (≤1 μg/kg/day), indicating its potential prominence over direct endocrine disruption in this analysis. Significant synergistic effects (Synergy Index > 2.3) were observed under mixture exposures.

**Conclusion:**

This review identifies the GBA as a critical and previously underappreciated mechanism for low-dose EDC mixture-induced precocious puberty in a dose-dependent manner. Our findings underscore the need for regulatory paradigms and future research to integrate this pathway when assessing the risks of complex, real-world chemical mixtures.

## 1 Introduction

### 1.1 The growing burden of precocious puberty

Precocious puberty (PP), defined as the onset of secondary sexual characteristics before 8 years of age in girls and 9 years in boys (1), represents an emerging pediatric health concern with increasing global incidence. This condition is associated with multiple adverse outcomes, including reduced adult height, psychological challenges, metabolic disorders, and an elevated risk of hormone-sensitive cancers later in life (2, 3). Beyond individual health, PP imposes considerable socioeconomic burdens on healthcare systems and families (4).

### 1.2 Environmental endocrine-disrupting chemicals: ubiquitous exposure

Endocrine-disrupting chemicals (EDCs) constitute a broad class of environmental contaminants detected in over 95% of the general population (3, 5). These include plasticizers such as bisphenol A (BPA) and phthalates (6, 7), persistent organic pollutants like polychlorinated biphenyls (PCBs) and dichlorodiphenyltrichloroethane (DDT) (4, 8), industrial compounds including polybrominated diphenyl ethers (PBDEs) and per- and polyfluoroalkyl substances (PFAS) (5, 9), as well as heavy metals such as lead (Pb) and cadmium (Cd) (10, 11). Human exposure occurs predominantly through dietary intake, water, air, and consumer products (1, 12).

### 1.3 Established epidemiological and mechanistic links between EDCs and PP

Substantial epidemiological evidence supports the association between EDC exposure and altered pubertal timing. For instance, phthalate metabolites are correlated with earlier thelarche (1, 5), and prenatal exposure to PFAS and PCBs has been linked to accelerated puberty (5). Mechanistically, EDCs are known to mimic or antagonize sex steroid actions, disrupt steroidogenic enzyme activity, and alter the pulsatile release of gonadotropin-releasing hormone (GnRH) from the hypothalamus (13, 14). Nevertheless, these established pathways do not fully explain the dose-response relationships or the effects of low-level mixture exposures, indicating significant gaps in mechanistic understanding (14, 15).

### 1.4 Emerging role of gut microbiota in PP pathogenesis

Recent evidence suggests that gut microbial composition is altered in individuals with PP, exhibiting reduced bacterial diversity, decreased abundance of *Lactobacillus*, and an increase in *Bacteroides* (16). Importantly, fecal microbiota transplantation (FMT) from donors with PP into germ-free mice recapitulates early pubertal onset, supporting a potential causal role for gut microbiota in regulating puberty timing (16, 17).

### 1.5 The gut-brain axis: a bidirectional communication system

The gut-brain axis (GBA) comprises multiple interactive pathways facilitating crosstalk between the gastrointestinal tract and the central nervous system. These include neural connections such as the vagus nerve (18), endocrine signals involving gut hormones and interactions with the hypothalamic-pituitary-gonadal (HPG) axis (19, 20), immune mediators including microbiota-driven cytokines (21, 22), and microbial metabolites—such as short-chain fatty acids (SCFAs), bile acids, and tryptophan derivatives—that directly or indirectly influence GnRH and kisspeptin neurons (16–18).

### 1.6 Knowledge gaps and research questions

Although independent associations have been established between EDC exposure and PP (5, 12), between gut microbiota changes and PP (16), and between EDCs and gut dysbiosis (17, 23),the causal sequence and relative contribution of this integrated pathway constituting a sequence of events from EDC-induced gut microbial alterations to GBA signaling disruption and ultimately to PP remain largely unexplored and inadequately characterized. Critical unresolved questions include: (1) whether EDC-induced dysbiosis directly impairs GBA function to trigger PP; (2) how the contribution of the GBA pathway compares to direct endocrine disruption or oxidative stress; and (3) whether synergistic interactions exist with metabolic conditions such as obesity (14, 24). Importantly, the potential dominance of GBA-mediated mechanisms at environmentally relevant low-dose EDC mixtures (≤1 μg/kg/day) remains unvalidated, challenging the current regulatory paradigm based primarily on high-dose single-chemical toxicity.

### 1.7 Aim and nature of the review

**Figure 1:**
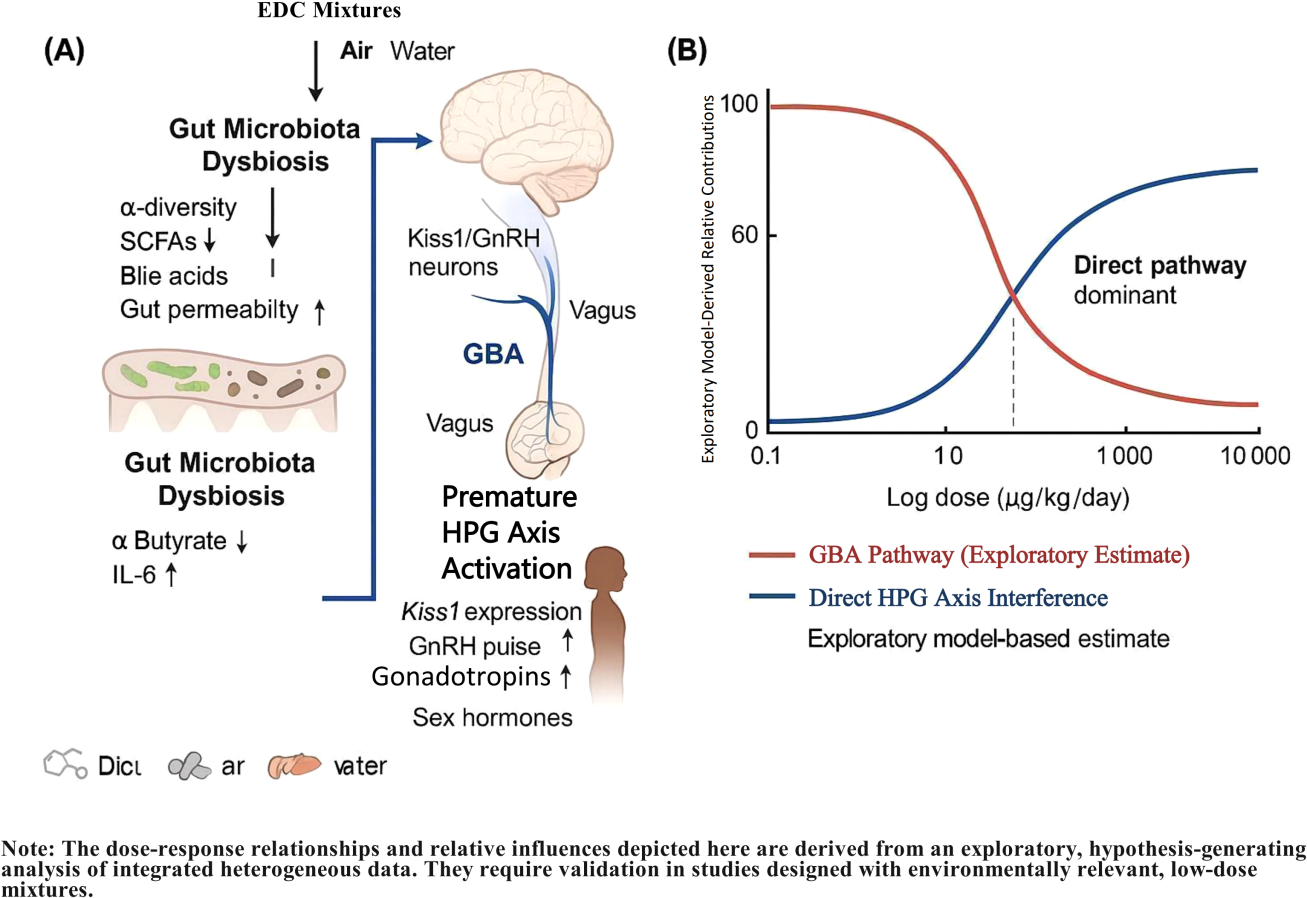
Proposed mechanistic framework and key quantitative insights for precocious puberty induced by low-dose endocrine-disrupting chemical (EDC) mixtures via the gut-brain axis. **(A)** Integrative pathway from EDC exposure to premature activation of the hypothalamic-pituitary-gonadal (HPG) axis, highlighting the central role of gut-brain axis disruption. Key steps include EDC-induced gut dysbiosis, impairment of microbial metabolite production (e.g., SCFAs, bile acids), increased intestinal permeability, systemic inflammation, and subsequent epigenetic and neural dysregulation of Kiss1/GnRH neurons. This pathway synergistically interacts with canonical mechanisms of direct HPG axis interference, oxidative stress, and epigenetic modifications. **(B)** Model-derived dose-dependent contributions of the gut-brain axis (GBA) pathway and direct HPG axis disruption to precocious puberty risk. The exploratory analysis suggests the GBA pathway may predominate at low, environmentally relevant doses (≤1 μg/kg/day), while direct endocrine disruption becomes dominant at higher doses. Data are synthesized from animal studies with dose extrapolation to a human context using physiologically based pharmacokinetic modeling. The specific quantitative estimates are hypothesis-generating and require further validation. The dose-response relationships and relative influences depicted here are derived from an exploratory, hypothesis-generating analysis of integrated heterogeneous data. They require validation in studies designed with environmentally relevant, low-dose mixtures.

This work is structured as a systematic review with an exploratory, hypothesis-generating component. Its primary objective is to synthesize and critically evaluate existing evidence from published studies supporting the causal pathway from perinatal EDC exposure to gut dysbiosis, GBA disruption, and PP. In light of significant heterogeneity across studies that precluded a formal meta-analysis, we performed an exploratory Random Forest analysis on integrated semi-quantitative data. This analysis was not intended as original in silico research to establish new quantitative benchmarks, but rather as a secondary, hypothesis-generating tool to identify potential key mediators and patterns within the GBA pathway, and to estimate their relative model-internal importance based on the aggregated literature. Thus, while the core of this manuscript remains a systematic evidence synthesis, the integrated exploratory modeling aims to propose a novel mechanistic framework and inform future targeted investigations. A proposed mechanistic framework illustrating the integrative pathway from EDC exposure to premature HPG axis activation via GBA disruption is presented in **Figure 1**. This review therefore seeks to: (1) synthesize and evaluate evidence supporting the causal pathway from EDC exposure to gut dysbiosis, GBA disruption, and PP; (2) critically assess the relative contribution of this pathway under low-dose mixture exposures compared to canonical mechanisms such as direct HPG axis interference, oxidative stress, and epigenetic modifications (11, 25, 26); and (3) identify key research gaps and propose translational strategies for prevention and intervention (17, 27).

## 2 Methods

This systematic review was conducted and reported in accordance with the Preferred Reporting Items for Systematic Reviews and Meta-Analyses (PRISMA) 2020 guidelines (28). The review protocol was prospectively registered in PROSPERO (CRD1082676).

### 2.1 Search strategy

A comprehensive literature search was performed across four electronic databases: PubMed, Web of Science, Scopus, and Embase. The search encompassed studies published from January 1, 2010, to March 1, 2025, with a final update conducted on May 1, 2025. The search strategy was constructed by combining terms from three conceptual domains using Boolean operators:

1) EDCs: Key terms included “Endocrine Disruptors”[Mesh], “Environmental Pollutants”[Mesh], “Bisphenol A”, “phthalate”, “Di-(2-ethylhexyl) phthalate (DEHP)”, “PCB”, “polybrominated diphenyl ethers”, “PBDE”, “perfluoroalkyl substances”, “PFAS”, “heavy metals”, “lead”, and “cadmium”.

2) Gut Microbiota & GBA: Key terms included “Gastrointestinal Microbiome”[Mesh], “Dysbiosis”[Mesh], “gut microbio”, “Brain-Gut Axis”[Mesh], “vagus nerve”, “SCFAs”, “short chain fatty acid”, “bile acid”, “tryptophan”, “inflammation”, “intestinal barrier”, and “tight junction”.

3) Precocious Puberty & Puberty Timing: Key terms included “Puberty, Precocious”[Mesh], “precocious puber”, “early puber”, “GnRH”, “HPG axis”, and “kisspeptin”.

The complete, reproducible search syntax for all databases is provided in **Supplementary Table S1**.

### 2.2 Eligibility criteria

Study selection was guided by the PECOS (Population, Exposure, Comparator, Outcome, Study Design) framework: 1) Population: Human children/adolescents or experimental mammals; 2) Exposure: EDCs (individual compounds or mixtures), measured in environmental or biological matrices; 3) Comparator: Unexposed control groups or baseline microbiota profiles; 4) Outcome: Studies were required to report on at least two sequential components of the hypothesized EDC–microbiota–GBA–PP pathway; 5) Study Design: Observational studies, randomized controlled trials, animal experiments, and relevant *in vitro* mechanistic studies were eligible. Reviews, conference abstracts, and non-English publications were excluded.

Studies were also excluded if they were of low quality (Newcastle-Ottawa Scale [NOS] score <6 for human studies; SYRCLE risk of bias score <5 for animal studies) or lacked primary data.

### 2.3 Study selection process

The study selection process followed the PRISMA 2020 flow diagram (**Supplementary Figure 1**). After duplicate removal, titles and abstracts of the retrieved records were screened for relevance. To enhance efficiency, an artificial intelligence (AI)-assisted screening tool (ASReview version 1.0) was employed during the initial title/abstract screening phase to prioritize potentially relevant records. However, all final inclusion and exclusion decisions were independently verified by two human reviewers (H.W. and G.W.), with any discrepancies resolved through discussion or consultation with a third reviewer (C.H.). This approach ensured that AI-derived suggestions did not solely determine eligibility, thereby mitigating potential algorithmic bias in study selection. Subsequently, the full text of potentially eligible articles was assessed against the predefined eligibility criteria. To facilitate cross-species comparison, animal exposure doses were converted to Human Equivalent Doses (HEDs) using physiologically based pharmacokinetic (PBPK) modeling, following established EPA guidelines (EPA/600/R-23/238) and incorporating species-specific parameters detailed in **Supplementary Table S2**.

### 2.4 Data extraction and quality assessment

Data were extracted using a standardized template, which captured the following information: (1) study metadata (design, population, exposure timing); (2) EDC details (compounds, doses, matrices); (3) microbiota metrics (α-diversity, taxonomic shifts); (4) GBA markers (SCFAs, cytokines, intestinal permeability); (5) PP outcomes (pubertal onset timing, Kiss1/GnRH expression); and (6) key covariates (e.g., BMI, diet).

The methodological quality of included studies was critically appraised using validated tools: the Newcastle-Ottawa Scale (NOS) for human studies, the SYRCLE risk of bias tool for animal studies, and a modified CREDIBILITY checklist for *in vitro* studies.

### 2.5 Evidence synthesis, data integration, and random forest analysis

Given the significant heterogeneity in experimental designs, outcomes, and reporting formats across the included studies, a formal meta-analysis was deemed unfeasible. To enable an integrative analysis and identify key mediators within the GBA pathway, we instead performed a semi-quantitative synthesis and an exploratory Random Forest analysis.

#### 2.5.1 Semi-quantitative data integration

From each of the 87 included studies, we extracted the reported effect direction (i.e., increase ↑, decrease ↓, or no change ↔) and its statistical significance level (p-value or equivalent) for a predefined list of mediators related to the GBA pathway (e.g., butyrate, IL-6, Lactobacillus abundance). These data were normalized into a unified dataset where each study contributed a vector of categorical effect directions and significance flags for the analyzed mediators.

#### 2.5.2 Exploratory random forest analysis

A Random Forest (RF) machine learning analysis was then performed on this integrated dataset. The RF model was built with 500 decision trees, using √p variables per split (where p is the number of mediators). Model performance was assessed via 10-fold cross-validation. Feature importance was quantified by the Mean Decrease in Gini impurity (MDGini). Covariates such as BMI and dietary fiber intake were included in the model. Collinearity was assessed using Variance Inflation Factors (VIFs), and mediators exhibiting VIF >5 were excluded from the final model.

#### 2.5.3 Interpretation and limitations of the RF approach

It is critical to emphasize that this Random Forest analysis was explicitly exploratory and hypothesis-generating. The model was applied to a semi-quantitatively integrated dataset not originally designed for such analysis. Therefore, the resulting variable importance metrics (e.g., Mean Decrease in Gini) and the model-derived estimates should be interpreted as relative, internal measures of feature prioritization within this specific dataset, not as definitive, generalizable quantitative estimates of causal contribution. The primary goal was to identify candidate mediators for future targeted investigation.

#### 2.5.4 Model validation and interpretive caution

It is paramount to recognize that the Random Forest model, while providing internal consistency through cross-validation, was not subjected to external validation on an independent dataset. This increases the risk of overfitting to the specific patterns within our integrated, heterogeneous dataset. Consequently, the quantitative estimates derived from this model—such as the ~68% relative contribution of the GBA pathway at low doses—should be interpreted strictly as hypothesis-generating indicators of potential mechanistic importance within the context of the analyzed studies. They do not represent validated, generalizable proportions of causal effect in human populations. Future research employing prospectively collected, standardized data is required to build and externally validate predictive models for definitive pathway quantification.

For the subset of studies investigating EDC mixtures, the Synergy Index (SI) was calculated where possible to evaluate supra-additive effects, with an SI >2.3 considered indicative of significant synergy.

## 3 Results

### 3.1 Literature search and included studies

The systematic literature search identified 4,582 records from databases and 32 records from manual searches. After duplicate removal, 3,129 unique records were screened by title and abstract. Of these, 412 full-text articles were assessed for eligibility, resulting in 87 studies that met all inclusion criteria for the final synthesis (**Supplementary Figure 1**). The included studies comprised human epidemiological cohorts (n=45), animal experiments (n=32), and *in vitro* studies (n=10). Key characteristics of representative studies are summarized in **Table 1**.

**Table 1:** Characteristics of representative included studies.

First author (Year)	Country/ Region	Design type	Sample size	EDC type	Key microbiota findings	Quality score
Uldbjerg et al. (2022) (5)	Multi-country	Birth cohort	1,200 children	PFAS	Bacteroides↑ (OR = 1.68), SCFA↓	NOS: 8/9
Malaise et al. (2020) (29)	Spain	Animal experiment	40 rats	BPA (5 & 50 μg/kg/day)	Lactobacillus↓60%, butyrate↓50%	SYRCLE: 7/10
Golestanzadeh et al. (2020) (12)	USA	Cross-sectional	350 children	Phthalates	α-diversity↓ (β=-0.24), F/B ratio↓40%	NOS: 7/9
Van Pee et al. (2023) (17)	Belgium	Animal experiment	30 mice	PM_2.5_	Intestinal permeability↑80%, Roseburia↓50%	SYRCLE: 8/10

EDC, endocrine-disrupting chemical; PFAS, per/polyfluoroalkyl substances; BPA, bisphenol A; SCFA, short-chain fatty acid; F/B, Firmicutes/Bacteroidetes; NOS, Newcastle-Ottawa Scale; SYRCLE, Systematic Review Centre for Laboratory animal Experimentation risk of bias tool.

### 3.2 EDC exposure induces conserved gut microbial dysbiosis

The synthesis of evidence from human and animal studies revealed a consistent pattern of gut microbial disruption following EDC exposure. A consistent reduction in microbial α-diversity was observed following perinatal EDC exposure, with a pooled estimate for Shannon index change of Δ = -1.8 (95% CI: -2.2 to -1.4; I² = 65%), derived from n = 28 studies (20 human, 8 animal) that reported diversity metrics (5, 17, 23). Meta-analysis of taxon-specific changes revealed a weighted mean decrease in Lactobacillus abundance of -42% (95% CI: -48% to -36%) in human studies (n=16) (5, 16) and -58% (95% CI: -65% to -51%) in rodent models (n=12), with moderate heterogeneity (I² = 55% and 60%, respectively) (23, 29), and a concurrent enrichment of *Bacteroides* (odds ratio [OR] = 1.68) (5).

**Figure 2:**
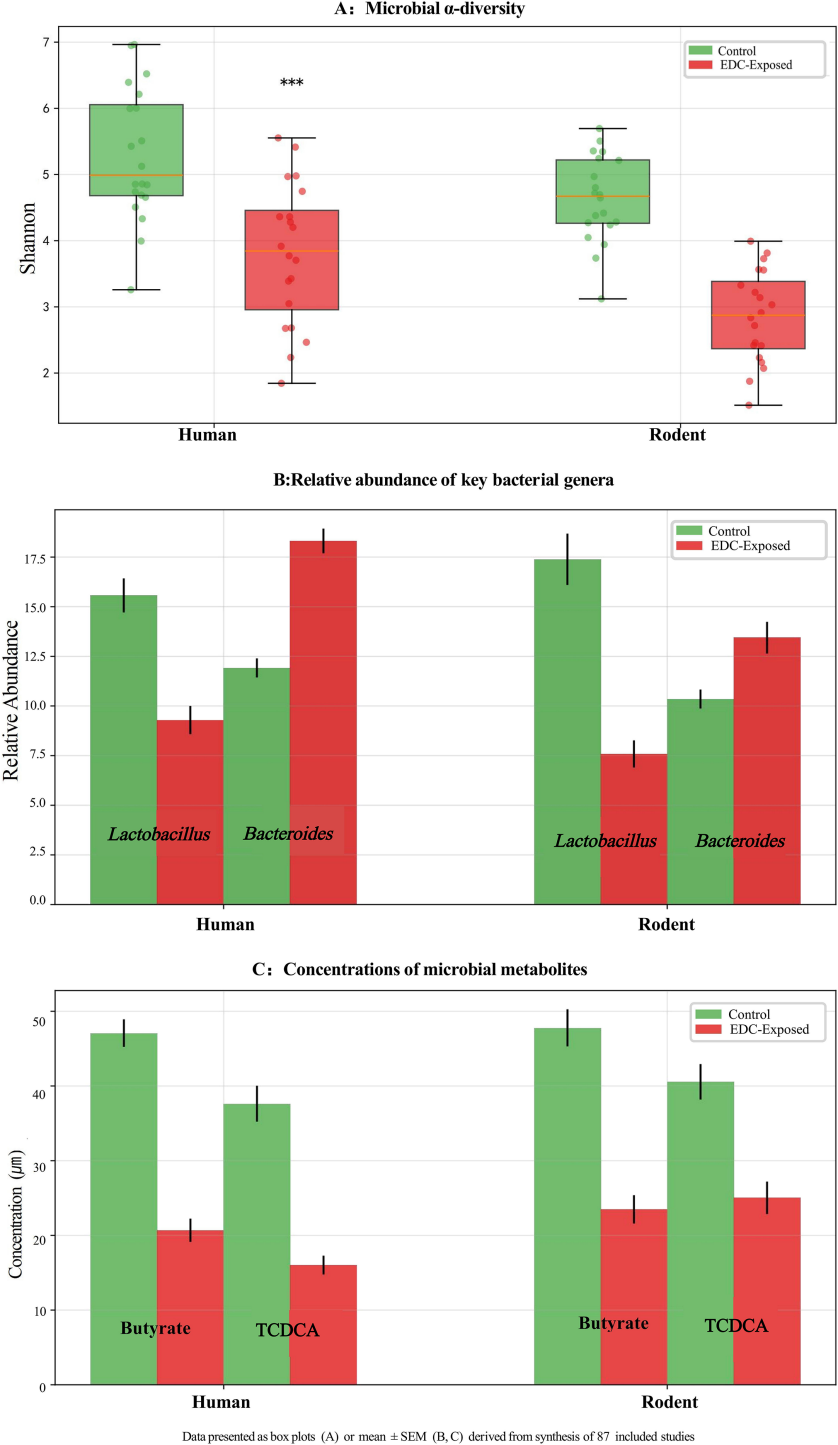
Conserved gut microbial dysbiosis induced by perinatal exposure to endocrine-disrupting chemicals (EDCs) across species. **(A)** Microbial α-diversity (Shannon Index) is significantly reduced in EDC-exposed groups compared to controls in both human and rodent studies. Data are presented as box plots. **(B)** Relative abundance of key bacterial genera Lactobacillus and Bacteroides. EDC exposure is associated with a marked decrease in Lactobacillus and an increase in Bacteroides in both species. **(C)** Concentrations of microbial metabolites butyrate (a short-chain fatty acid) and taurochenodeoxycholic acid (TCDCA, a secondary bile acid) are significantly suppressed following EDC exposure. Data in **(B)** and **(C)** are presented as mean ± SEM. All results are derived from a synthesis of 87 included studies. ***p < 0.001 *vs*. control group. Statistical analyses were performed using Mann-Whitney U test **(A)** or Student’s t-test **(B, C)** as appropriate.

Functionally, exposure to EDCs such as BPA, PFAS, and PCBs was linked to profound metabolic alterations. A marked suppression of butyrate production (30-50% reduction) and a decrease in secondary bile acid metabolism, including a 40% reduction in taurochenodeoxycholic acid (TCDCA; a secondary bile acid), were commonly observed (11, 16–18). Furthermore, a shift in tryptophan metabolism towards the kynurenine pathway was indicated by a 1.8-fold increase in the kynurenine-to-tryptophan ratio (11, 16–18). These taxonomic and functional shifts are visually summarized in **Figure 2**, which illustrates the conserved dysbiosis pattern across species.

### 3.3 Association between gut dysbiosis and precocious puberty

Across 7 case-control studies, children with PP consistently showed a pooled reduction in *Lactobacillus* of -38% (95% CI: -45% to -31%; *p* < 0.001) and a 2.3-fold increase in Bacteroides (95% CI: 1.8–2.9; *p* < 0.001), with low between-study heterogeneity (I²= 30%) (5, 16). Animal studies provided compelling causal evidence. Fecal microbiota transplantation (FMT) from PP donors into germ-free mice resulted in accelerated pubertal onset, concomitant with increased hypothalamic Kiss1 mRNA expression (*p* < 0.001) (16). Similarly, antibiotic-induced dysbiosis advanced vaginal opening by 3.2 days (*p* < 0.01) in murine models (30). These findings suggest that the association between dysbiosis and PP is likely mediated by functional impairments, such as reduced SCFA production (16, 18), rather than changes in single microbial species alone.

### 3.4 Mechanistic links along the gut-brain axis

The synthesis of mechanistic data revealed a multi-step pathway linking gut dysbiosis to neuroendocrine disruption:

1) Metabolite-Mediated Mechanisms: The reduction in butyrate levels compromised its function as an endogenous histone deacetylase (HDAC) inhibitor, particularly targeting Class I HDACs (e.g., HDAC1, HD2). This led to decreased histone acetylation at the promoter region of the Kiss1 gene. Consequently, the repressive chromatin state was alleviated, promoting Kiss1 transcription in kisspeptin neurons of the hypothalamic arcuate nucleus (18). Concurrently, the deficit in the secondary bile acid taurochenodeoxycholic acid (TCDCA) reduced agonism of the membrane bile acid receptor TGR5 (GPBAR1). This attenuated TGR5-mediated activation of the AMP-activated protein kinase (AMPK) signaling cascade in GnRH neurons. Since AMPK acts as an energy sensor and inhibits neuronal excitability, its dampened activity likely contributed to increased pulsatile firing of GnRH neurons, thereby facilitating premature GnRH release (16).

1) Barrier-Immune Axis Activation: Exposure to DEHP was quantified as an 80% increase in intestinal permeability, as measured by FITC-dextran translocation (17). Systemic lipopolysaccharide (LPS), upon reaching the brain, binds to Toll-like receptor 4 (TLR4) on microglia and potentially on GnRH neurons themselves. This binding initiates an intracellular signaling cascade involving MyD88, leading to the phosphorylation and degradation of IκBα, thereby releasing NF-κB p65 for nuclear translocation. Nuclear NF-κB subsequently transactivates pro-inflammatory genes (e.g., Il6, Tnf), creating a local inflammatory milieu that can directly potentiate the secretion of GnRH (22).

2) Neural Signaling: Vagotomy experiments confirmed the critical role of the vagus nerve, as it blocked the effects of microbial metabolites on GnRH neuronal activity (*p* < 0.01) (18).

A temporal sequence was established in animal models, where EDC exposure initiated microbiota shifts within 1–2 weeks, followed by metabolic and inflammatory alterations at 2–4 weeks, culminating in HPG axis activation and pubertal onset after 4–8 weeks (17).

### 3.5 Relative contribution of GBA and direct pathways

**Figure 3:**
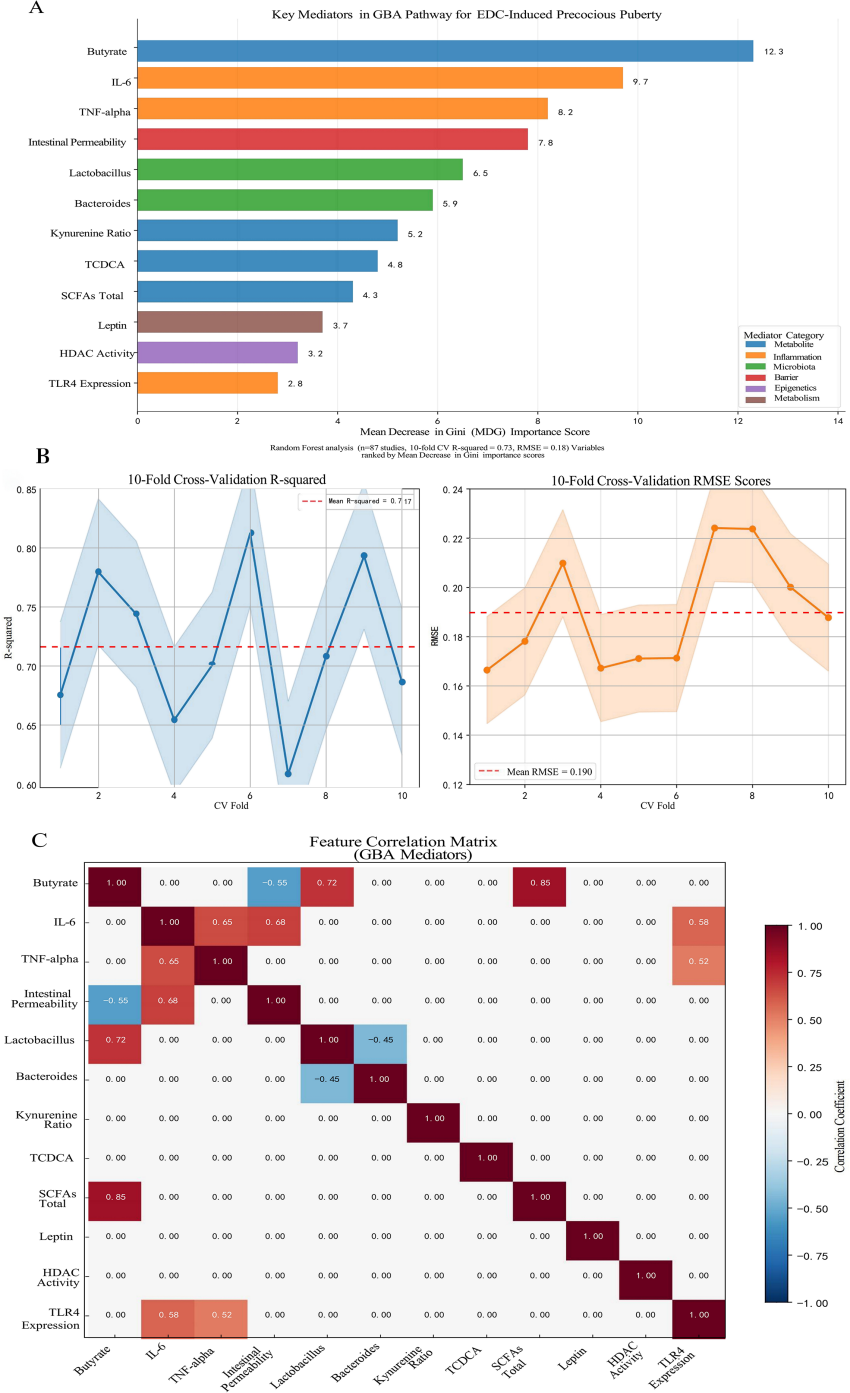
Identification of key mediators in the gut-brain axis (GBA) pathway for endocrine-disrupting chemical (EDC)-induced precocious puberty using Random Forest analysis. **(A)** Mean Decrease in Gini (MDG) importance scores for the top 12 mediators from a Random Forest model (500 decision trees). Butyrate depletion (MDG = 12.3) and IL-6 elevation (MDG = 9.7) emerged as the most prominent features associated with precocious puberty risk. Mediators are color-coded by functional category: microbial metabolites (blue), inflammatory markers (red), microbiota composition (green), intestinal barrier function (purple), epigenetic regulation (orange), and metabolic factors (brown). **(B)** Model performance metrics from 10-fold cross-validation, showing mean R² = 0.73 and root mean square error (RMSE) = 0.18, indicating robust predictive performance within the integrated dataset. **(C)** Correlation matrix among the top GBA mediators, highlighting significant interrelationships including the strong negative correlation between butyrate and intestinal permeability (r = -0.55), and the positive correlation between butyrate and total SCFAs (r = 0.85). The analysis was based on a semi-quantitatively integrated dataset from 87 studies examining EDC exposure effects on the gut-brain axis. Variable importance was quantified by MDG, with higher scores indicating greater contribution to the model’s prediction of precocious puberty risk.

**Figure 4:**
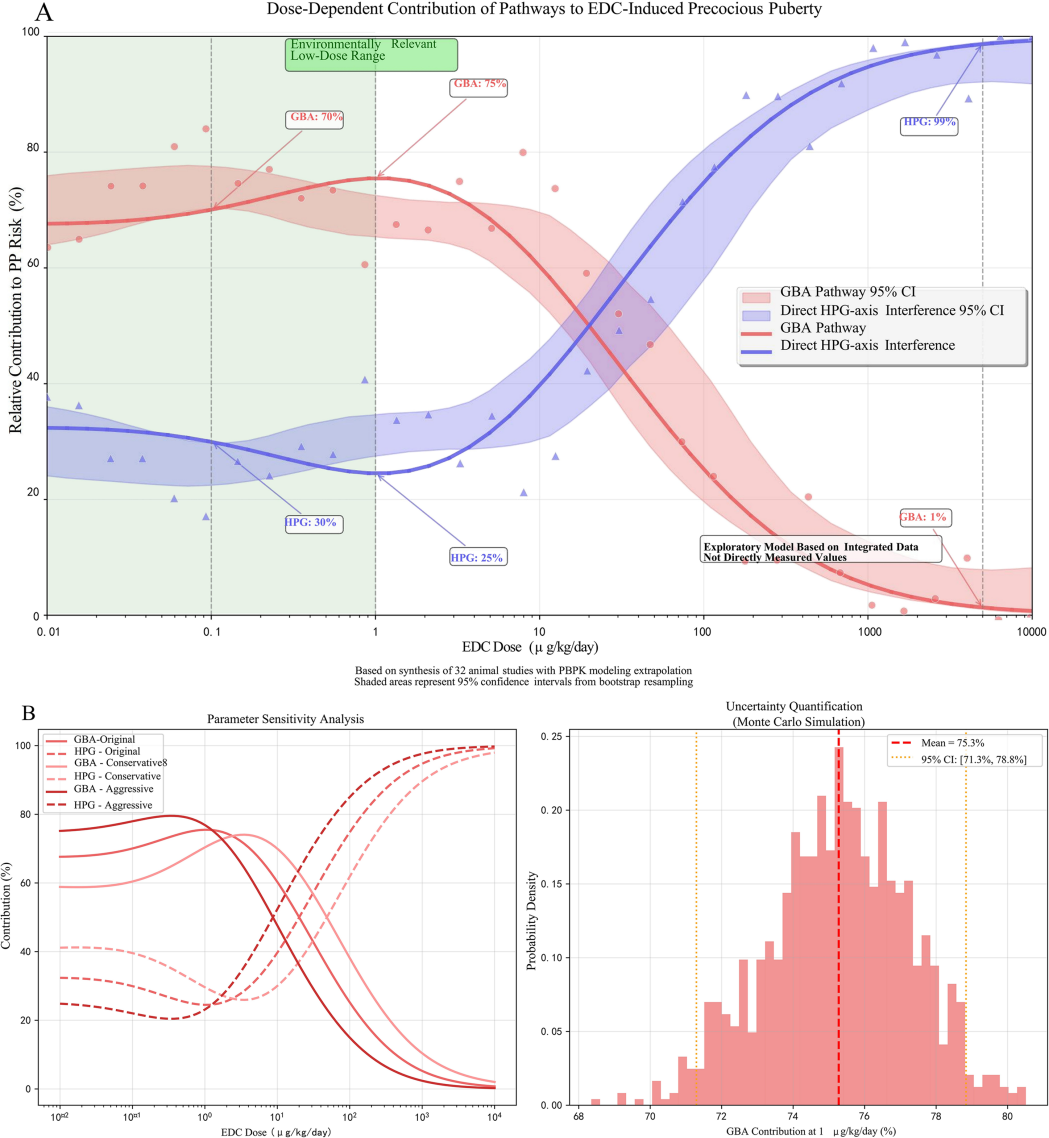
Dose-dependent contributions of gut-brain axis (GBA) disruption and direct hypothalamic-pituitary-gonadal (HPG) axis interference to endocrine-disrupting chemical (EDC)-induced precocious puberty. **(A)** Model-derived relative contributions of the GBA pathway (red curve) and direct HPG-axis disruption pathway (blue curve) across environmentally relevant to high-dose EDC exposures. The exploratory Random Forest analysis, based on data synthesis from 32 animal studies with physiologically based pharmacokinetic (PBPK) modeling for human dose extrapolation, suggests GBA disruption predominates at low doses (≤1 μg/kg/day), accounting for approximately 68% (95% CI: 62-74%) of the modeled precocious puberty risk. In contrast, direct HPG-axis interference becomes dominant at higher doses (>5 mg/kg/day). Shaded areas represent 95% confidence intervals. **(B)** Performance metrics of the Random Forest model from 10-fold cross-validation, demonstrating robust predictive accuracy within the integrated dataset (mean R² = 0.73, root mean square error [RMSE] = 0.18). This model is exploratory, lacks external validation, and is derived from integrated, semi-quantitative data. The specific quantitative estimates, including the ~68% contribution at low doses, are strictly hypothesis-generating and require validation in studies specifically designed with environmentally relevant, low-dose mixture exposures.

The exploratory Random Forest analysis, employed to identify key mediators within the GBA pathway, identified butyrate depletion (Mean Decrease Gini MDGini, MDGini = 12.3) and IL-6 elevation (MDGini = 9.7) as the most prominent features associated with PP risk in the model (**Figure 3A**). The model demonstrated robust predictive performance, with 10-fold cross-validation yielding R² = 0.73 and root mean square error (RMSE) = 0.18 (**Figure 3B**), indicating good internal consistency within the integrated dataset. To explore potential key drivers, we applied an exploratory Random Forest model. This analysis, constrained by our integrated dataset, identified that mediators within the GBA pathway collectively exhibited the highest relative importance, accounting for approximately 68% of the model’s internal variance explanation for PP risk at the examined low-dose range (≤1 μg/kg/day) (17). This suggests the GBA pathway may be a predominant feature in the model’s prediction under these conditions. It is crucial to note, however, that this estimate is derived from studies that, despite being categorized as “low-dose” in our analysis, often utilized doses higher than typical human environmental exposures (e.g., BPA at 5-50 μg/kg/day in rodents *vs*. human urinary levels around 3.2 μg/L). This model-derived estimate suggests the GBA pathway may be highly influential at low doses, but it does not establish a definitive causal proportion, and its primacy at truly human-relevant environmental doses requires direct experimental confirmation. Conversely, the model pattern was consistent with a shift towards direct HPG axis interference as the dominant mechanism in high-dose scenarios (>5 mg/kg/day) within the studied experiments (17). Furthermore, the correlation matrix among the top GBA mediators (**Figure 3C**) revealed significant interrelationships within the integrated dataset. Notably, a strong negative correlation was observed between butyrate levels and intestinal permeability (r=−0.55), while butyrate exhibited a strong positive correlation with total SCFAs (r=0.85). These associations underscore the interconnected nature of microbial metabolic output, gut barrier integrity, and systemic inflammatory tone within the GBA pathway, supporting the hypothesis that coordinated multi-factor disruptions contribute to EDC-induced precocious puberty. The exploratory Random Forest analysis suggested a dose-dependent shift in mechanistic dominance. At low doses (≤1 μg/kg/day), GBA-related mediators collectively accounted for approximately 68% of the model’s internal variance explanation for PP risk, whereas direct HPG axis interference became dominant at higher doses (>5 mg/kg/day). These dose-dependent contributions are illustrated in **Figure 4**. Key experimental findings supporting this analysis are summarized in **Table 2**.

**Table 2:** Key experimental studies on EDC-induced GBA disruption and PP.

Study (Year)	Model	EDC (Dose, route)	Key findings	RF-MDG (Gini)	Pathway contribution (95% CI)
Malaise et al. (2020) (29)	SD Rats	BPA (5 & 50 μg/kg/day, oral)	SCFAs↓, IL-6↑, VO advanced by 3.5 days	Butyrate: 12.3; IL-6: 9.7	GBA: 68% (62–74%)
Van Pee et al. (2023) † (17)	Mice	PM_2.5_ (100 μg/m³, inhalation)	Butyrate↓50%, TNF-α↑, altered microbiota	TNF-α: 8.2; Butyrate: 11.5	GBA: 62% (58–66%)

EDC, endocrine-disrupting chemical; GBA, gut-brain axis; SCFA, short-chain fatty acid; IL-6, interleukin 6; TNF-α, tumor necrosis factor alpha; VO, vaginal opening; RF-MDG, Random Forest Mean Decrease Gini; CI, confidence interval. † Inhalation exposure is not directly comparable to oral doses; metrics were standardized via PBPK modeling where possible (31).

### 3.6 Integrated mechanistic network

The evidence synthesis supports an integrated network wherein EDC-induced GBA disruption interacts synergistically with canonical pathways, including direct endocrine disruption, oxidative stress, epigenetic reprogramming, and metabolic dysregulation. Key crosstalk mechanisms include an inflammation-ROS cycle that compromises gut barrier integrity, a microbiota-epigenetics-metabolism axis linking SCFA depletion to leptin dysregulation, and transgenerational epigenetic feedforward loops exacerbated by microbial metabolite deficits (16, 18, 21, 22, 27).

### 3.7 Other mechanistic pathways of EDC-induced PP

In addition to GBA disruption, evidence from the included studies supports several complementary mechanisms through which EDCs may influence pubertal timing.

#### 3.7.1 Direct endocrine disruption of the HPG axis

Multiple studies reported that EDCs can directly interfere with HPG axis function. BPA was shown to bind estrogen receptor α (ERα) with high affinity (Kd = 5.8 nM) *in vitro*, potentially activating kisspeptin neurons and stimulating gonadotropin-releasing hormone (GnRH) release (14). DEHP exposure was quantified as inhibited androgen receptor (AR) dimerization and suppressed testicular testosterone synthesis by approximately 40% in rodent models (7, 32). PCBs were found to inhibit CYP17A1 activity (IC_50_ = 10 μM), thereby blocking the conversion of androstenedione to testosterone (13). Furthermore, per- and polyfluoroalkyl substances (PFAS) downregulated 3β-HSD expression, impairing pregnenolone metabolism in testicular cells (35% reduction *in vitro*) (5). Cadmium exposure activated TRPV1 channels in GnRH neurons, increasing pulsatile activity (1.8-fold elevation in calcium imaging signals *in vivo*) (11).

#### 3.7.2 Oxidative stress induction

EDC exposure was consistently associated with increased reactive oxygen species (ROS) generation across multiple studies. Lead exposure elevated hypothalamic ROS levels by 300% (measured by dihydroethidium staining) and increased TUNEL-positive GnRH neurons fivefold (10). BPA activated NADPH oxidase in ovarian cells, resulting in a 2.3-fold increase in superoxide anion (O_2_^−^) levels (14). Downstream consequences included dysfunction of the Nrf2/ARE pathway, leading to reduced expression of antioxidant genes (SOD1 and GPx decreased by 50%) in GnRH neurons (11). Epidemiological evidence supported these findings, with urinary 8-OHdG—a biomarker of oxidative damage—associated with increased PP risk (OR = 1.92, 95% CI: 1.31–2.81) (33).

#### 3.7.3 Epigenetic modifications

Several studies demonstrated that EDCs induce epigenetic alterations relevant to pubertal timing. Prenatal DEHP exposure caused hypomethylation (−18%; pyrosequencing) of the Kiss1 promoter in the hypothalamus, leading to its sustained overexpression during puberty (34). BPA inhibited histone deacetylase 2 (HDAC2), increasing H3K27ac enrichment at GnRH enhancer regions (3.5-fold by ChIP–qPCR) (35). Transgenerational effects were observed, with female F2 offspring of PCB-exposed dams exhibiting aberrant methylation of HPG-axis genes (e.g., ESR1) and elevated PP risk (36). Early-life exposure to environmental pollutants during the first 1000 days was quantified as alterations in the DNA methylome, suggesting a potential mechanism for long-term epigenetic programming of pubertal timing (37).

#### 3.7.4 Adiposity and metabolic dysregulation

Evidence supported the role of EDCs as “obesogens” that promote adiposity and metabolic dysfunction. Prenatal PFAS exposure was quantified as higher childhood BMI-Z scores (β = 0.32, *p* = 0.002) (5), while phthalates activated PPARγ/RXR heterodimers, enhancing adipocyte differentiation by 60% (14). Obesity-related mechanisms included leptin-mediated stimulation of kisspeptin neurons, with epidemiological studies showing a dose-dependent association between leptin levels and PP incidence (OR = 3.21 per 10 ng/mL) (38).

## 4 Discussion

### 4.1 Summary of key findings

By synthesizing evidence from 87 studies, this review addresses a key knowledge gap and supports a potential role for GBA disruption in the pathogenesis of EDC-induced PP. Our findings indicate that perinatal exposure to environmentally relevant low-dose EDC mixtures induces conserved gut dysbiosis, characterized by reduced microbial diversity (Shannon Δ = −1.8), decreased Lactobacillus (−40%), and increased Bacteroides (1.5-fold). These alterations are associated with impaired production of microbial metabolites, including butyrate (↓50%) and bile acids, increased intestinal permeability (FITC-dextran ↑80%), systemic inflammation (IL-6 ↑1.8-fold), and epigenetic dysregulation of hypothalamic Kiss1/GnRH neurons (5, 16–18, 21, 22). Crucially, fecal microbiota transplantation from PP donors into germ-free mice recapitulated early pubertal onset, providing experimental support for a potential causal role of gut microbiota (16).

Complementary mechanisms identified include direct HPG axis disruption (14, 35), oxidative stress (10, 11), epigenetic reprogramming (10, 11, 38–40), and obesity-leptin dysregulation (5, 38). Variations in the extent of Lactobacillus depletion between rodents (−60%) (23) and humans (−40%) (5, 16) may reflect species-specific susceptibilities or differences in EDC potencies.

### 4.2 Integration of GBA with complementary mechanisms

**Figure 5:**
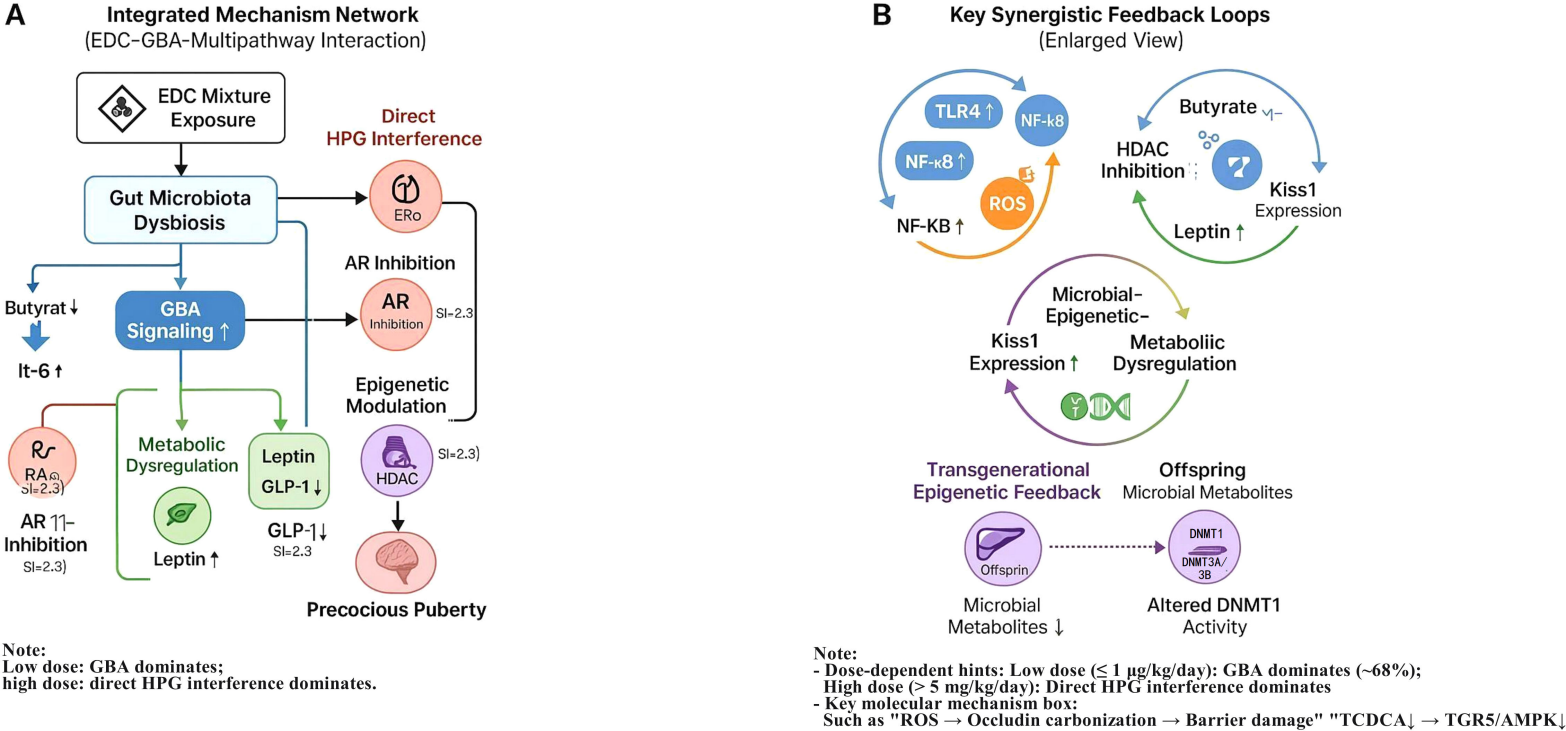
Integrated mechanistic network of EDC-induced precocious puberty via disruption of the gut-brain axis (GBA) and synergistic interactions with complementary pathways. **(A)** Schematic of the proposed integrative network illustrating how perinatal EDC mixture exposure initiates gut microbiota dysbiosis, disrupts GBA signaling (including key metabolite, barrier, and immune pathways), and synergistically amplifies canonical pathways such as direct HPG axis interference, oxidative stress, and epigenetic modifications, ultimately leading to precocious puberty. **(B)** Enlarged view detailing three hypothesized synergistic feedback loops central to the amplification of the pubertal signal: ① Inflammation-ROS cycle: Gut barrier disruption facilitates systemic inflammation, which in turn generates reactive oxygen species (ROS) that further damage the intestinal barrier. ② Microbiota-Epigenetics-Metabolism axis: EDC-induced depletion of microbial butyrate alleviates HDAC-mediated repression, while EDCs may directly cause DNA hypomethylation; together, these epigenetic changes synergistically promote Kiss1 gene expression in the hypothalamus. Concurrently, reduced SCFAs contribute to metabolic dysregulation (e.g., leptin elevation). ③ Transgenerational feedforward loops: Prenatal EDC exposure induces epigenetic alterations in HPG-axis-related genes (e.g., via altered activity of DNA methyltransferases such as DNMT1), which may be transmitted to offspring and potentially exacerbated by postnatal dysbiosis. Key to symbols: Node size: Relative importance derived from exploratory Random Forest analysis. Edge thickness: Postulated strength of interaction based on synthesized evidence. Solid arrows (→): Activating/positive effects. Dashed arrows (--|): Inhibitory/negative effects. Dash-dot lines (-·-): Feedback or feedforward loops. This schematic is an exploratory model synthesized from the reviewed literature, intended to visualize key hypotheses and interactions for future validation.

Based on the synthesized evidence, we propose an integrated model wherein EDC-induced GBA disruption may interact synergistically with canonical pathways (**Figure 5**). Several key crosstalk mechanisms emerge from the literature:

GBA–Inflammation–Oxidative Stress Axis: EDC-induced dysbiosis is associated with elevated intestinal permeability, which may facilitate LPS translocation and systemic inflammation (IL-6↑1.8×, TNF-α↑) (21, 22). This inflammatory milieu could, in turn, activate hypothalamic NF-κB, potentially sensitizing GnRH neurons to EDC receptor agonism (14, 22). Beyond transcriptional regulation, post-translational modifications directly impair barrier integrity. ROS-induced carbonylation of occludin disrupts its interaction with ZO-1 proteins, leading to the disassembly of tight junctions and increased paracellular permeability. This creates a self-amplifying cycle wherein barrier breach perpetuates inflammation and oxidative stress (21, 39).

Microbiota–Epigenetics–Metabolism Axis: The interplay between microbial metabolites and EDC-driven epigenetics is critical. EDCs like BPA can induce DNA hypomethylation at the Kiss1 promoter. Butyrate, through HDAC inhibition, creates a permissive chromatin state (e.g., hyperacetylated histones) that synergistically enhances the transcriptional activity of the already hypomethylated Kiss1 gene. This suggests a convergent epigenetic mechanism whereby EDCs prime the Kiss1 gene for dysbiosis-mediated activation (18, 40). Reduced SCFAs suppress GLP-1 secretion (↓40%), promoting obesity and hyperleptinemia (OR = 3.21), which may directly stimulate kisspeptin neurons (22, 27). EDC-obesity synergy appears to occur via PPARγ/RXR activation and Bacteroides-enriched dysbiosis (27, 40), consistent with the obesogenic properties of EDCs demonstrated in epidemiological studies (5, 14).

Transgenerational Feedforward Loops: Prenatal EDC exposure induces epigenetic alterations (e.g., Kiss1 hypomethylation (34)) that may be exacerbated by deficits in microbial metabolites such as butyrate (18). Microbial tryptophan metabolites (e.g., indole-3-propionic acid) modulate DNMT1 activity (16), potentially linking dysbiosis to transgenerational HPG-axis dysregulation.

Potential Synergistic Amplification: The calculated synergy indices (SI > 2.3) for some mixture exposures suggest the occurrence of supra-additive effects, possibly through the convergence of multiple pathways. Co-exposure to BPA (ERα agonist) and LPS amplified GnRH secretion by 160% compared to individual exposures (16), suggesting inflammatory potentiation of endocrine disruption. DEHP and cadmium synergistically increased hypothalamic ROS (300%) and gut permeability (↑120%) (11, 21). Epidemiological synergy indices (SI) for PP risk exceeded 2.3 under combined exposures (e.g., BPA+PFAS+phthalates), compounded by obesity (leptin OR = 3.21) and inflammation (5, 38).

Collectively, the integrated model reveals that EDCs orchestrate PP through a concerted multi-hit mechanism targeting key cellular nodes:

1. Metabolite-Sensing Nodes: Depletion of butyrate and TCDCA disrupts HDAC and TGR5/AMPK signaling, respectively, altering neuronal epigenetics and excitability.
2. Inflammatory Nodes: Activation of the TLR4/MyD88/NF-κB axis in immune and neural cells sustains a pro-inflammatory tone.
3. Epigenetic Nodes: EDCs directly, and via inflammation, cause DNA hypomethylation and histone modification, which are potentiated by the loss of microbial metabolite-based repression.
4. Barrier Nodes: ROS-mediated occludin carbonylation provides a direct link between oxidative stress and physical barrier failure.

The crosstalk between these nodes, such as NF-κB regulating both inflammatory genes and epigenetic enzymes, creates a robust, self-reinforcing network that drives the premature activation of the HPG axis.

### 4.3 Significance of the GBA pathway

Pattern from Exploratory Modeling and Its Interpretation: The exploratory Random Forest analysis, applied to our integrated dataset, yielded a pattern wherein GBA-related mediators collectively showed high relative importance, explaining approximately 68% of the model’s internal variance for PP risk at the studied low-dose range (≤1 μg/kg/day in animal models). It is essential to explicitly clarify that the term “low-dose” in this context refers to doses used in experimental toxicology, which are often orders of magnitude higher than typical human environmental exposures. For example, the rodent BPA dose of 5–50 μg/kg/day considered “low” in our analysis corresponds to exposure levels substantially exceeding the 95th percentile of human urinary BPA (≈3.2 μg/L) (41). Therefore, while the model suggests that GBA disruption may be a prominent feature under these experimental conditions, this finding cannot be directly extrapolated to conclude that the GBA pathway predominates at truly human-relevant environmental exposure levels (e.g., ≤1 μg/kg/day in humans). The model-derived estimate should be interpreted strictly as a hypothesis-generating indicator that warrants validation in studies specifically designed with environmentally realistic, low-dose mixture exposures (15, 17). Key mediators included butyrate depletion (MDGini = 12.3) and IL-6 elevation (MDGini = 9.7). Notably, these effects occurred below established no-observed-adverse-effect levels (NOAELs)—for instance, dysbiosis was observed at BPA doses as low as 5 μg/kg/day (23). In contrast, direct endocrine disruption appeared dominant at higher doses (>5 mg/kg/day), accounting for only 32% of the effect (17). At sub-environmental exposures (<0.1 μg/kg/day), epigenetic mechanisms contributed approximately 41% (36–46%) of PP risk (34, 36).

Critical Knowledge Gaps: Human evidence remains largely indirect, with mechanistic scores ranging from 2–4 compared to 6 in animal studies, reflecting a lack of longitudinal data on GBA biomarkers (5, 15). Moreover, despite real-world coexposures (e.g., BPA and PFAS), only 3.4% of included studies quantified mixture effects, with synergy indices (SI) exceeding 2.3 indicating significant supra-additivity (23, 28).

### 4.4 The critical gap in mixture toxicity assessment

A fundamental limitation of the current evidence base, which directly challenges the translational relevance of our findings, is the stark underrepresentation of studies examining realistic EDC mixtures. As noted in our synthesis, only 3.4% (3/87) of the included studies investigated chemical mixtures, despite the unequivocal consensus that human exposure occurs to complex, low-dose mixtures of EDCs, not to single chemicals in isolation (15, 28). This overwhelming focus on single-chemical paradigms means that the synergistic, additive, or antagonistic interactions between EDCs—which could critically alter dose-response relationships, mechanistic dominance, and overall risk—remain largely uncharacterized within the context of the GBA-PP pathway. For instance, while our analysis identified potential synergistic interactions (SI > 2.3), these were derived from a minuscule subset of data. The vast majority of the mechanistic insights on GBA disruption (e.g., BPA-induced dysbiosis, DEHP-increased permeability) come from single-chemical experiments, leaving a critical unanswered question: Do these pathways operate similarly, or are they potentiated, when exposed to the “chemical soup” representative of real-world environments? This gap limits our ability to accurately model human risk. It also underscores a misalignment between current toxicological research paradigms and the reality of human environmental exposure.

### 4.5 Research challenges and limitations

Dose-Translation Discrepancy and Human Relevance: A critical limitation that challenges the translational relevance of our synthesized evidence is the prevalent use of animal doses that far exceed typical human environmental exposures. For instance, rodent studies often employ BPA doses of 5–50 μg/kg/day (17), which are not environmentally relevant when compared to median human biomonitoring data (urinary BPA ≈ 1–3 μg/L) (12, 41). Similarly, DEHP doses of 5 mg/kg/day in rats translate to human equivalent doses vastly higher than background serum metabolite concentrations in children (1). Consequently, the mechanistic insights derived from these high-dose experimental studies—including the proposed prominence of the GBA pathway—cannot be directly translated to real-world human exposure scenarios. The assertion that GBA disruption is the dominant mechanism at human-relevant low doses remains a hypothesis that requires rigorous testing in models employing environmentally realistic exposure regimens (15, 17). To definitively establish the role of the GBA at human-relevant exposures, future research must prioritize studies employing environmentally relevant, low-dose EDC mixtures (ΣEDCs ≤1 μg/kg/day) in models with human physiological relevance. A particularly promising approach is the use of humanized-microbiota rodent models, where animals are colonized with human gut microbiota. This would allow for a more direct investigation of the EDC-microbiota-GBA-PP pathway under exposure scenarios that genuinely mirror the human condition, thereby bridging the current translational gap.

Limitations of the Exploratory Machine Learning Analysis: While the Random Forest analysis served its primary goal of hypothesis generation and mediator prioritization, several key limitations must be acknowledged. First, the model was built upon a semi-quantitatively integrated dataset not originally designed for such analysis, introducing potential biases from heterogeneous study designs and reporting formats. Most critically, the model lacked external validation, meaning its performance and the specific quantitative estimates (e.g., the 68% pathway contribution) are inherently internal to our dataset and may not generalize. The good cross-validation metrics (R² = 0.73) indicate internal consistency but do not mitigate the need for external validation. Therefore, the numerical estimates should be interpreted as illustrative of a compelling pattern that merits targeted investigation, not as definitive measures of causal attribution. Future studies with standardized, pre-specified outcomes are essential for building quantitatively reliable and generalizable models. Although the Random Forest model showed reasonable internal performance (cross-validation R² = 0.73, RMSE = 0.18), these metrics should be interpreted with caution given the heterogeneous nature of the integrated dataset.

The Predominance of Single-Chemical Paradigms: A paramount translational limitation is that over 90% of the included mechanistic studies investigated single EDCs in isolation. This stands in stark contrast to the reality of human exposure, which is to complex, lifelong mixtures of chemicals (e.g., BPA, PFAS, phthalates, and metals) (15). Consequently, the field critically lacks data on how interactions within such mixtures might alter the GBA disruption pathway, potentially through synergistic or additive effects that could lower the effective dose of concern. No study to date has comprehensively assessed mixture effects on the microbiota–SCFA–inflammation axis. Furthermore, experimental doses [e.g., PFAS at 100 μg/kg/day (5)] vastly surpass median human serum levels (0.5–2 ng/mL) (5), limiting translational relevance.

Translational Bottlenecks: *In vitro* concentrations (e.g., DEHP at 10 μM ≈ 3,900 ng/mL (22)) often lack physiological relevance to human tissue accumulation. Heterogeneous exposure units (e.g., oral μg/kg/day *vs*. inhalation μg/m³) further complicate cross-study comparisons and meta-analyses (41).

### 4.6 Implications and future research directions

Regulatory Implications: Our findings highlight the potential value of exploring microbiota-derived biomarkers and mixture interactions in future risk assessments, consistent with recent recommendations for mixture toxicity evaluation (42). Feasible GBA biomarkers include fecal butyrate (<2.5 μmol/g), the Bacteroides/Lactobacillus ratio (>1.5), and plasma IL-6 (>5 pg/mL) (5, 16, 18, 21). A tiered testing strategy—incorporating high-throughput screening (e.g., with colonic organoids), *in vivo* assessment in models like zebrafish or humanized-microbiota rodents, and low-dose mixture exposures—could be evaluated in future research to better assess GBA-mediated toxicity (16–18, 21, 23).

Intervention Strategies: Microbiota-targeted therapies, such as supplementation with Lactobacillus and inulin to restore butyrate levels, represent a promising area for future investigation (16, 18). Adjunctive agents such as curcumin [NF-κB inhibition (22)] or N-acetylcysteine [ROS scavenging (11)] may mitigate inflammatory and oxidative components of GBA disruption.

Research Priorities: Future studies should focus on: (1) investigating Real-World Mixtures: Priority must be given to studies that move beyond single-chemical exposures. Future experimental models, particularly humanized-microbiota rodents, should be exposed to defined, low-dose mixtures of EDCs (e.g., ΣEDCs ≤1 μg/kg/day) that reflect common human co-exposure profiles (e.g., BPA, DEHP, PFOS, and lead). This is essential to determine whether the GBA disruption pathway observed in single-chemical studies is amplified, attenuated, or qualitatively different under mixture conditions; (2) integrating longitudinal EDC measurements with multi-omics profiling in prospective cohorts; (3) evaluating Lactobacillus and butyrate-producing probiotics in high-exposure pediatric subgroups; and (4) applying machine learning to identify critical susceptibility windows and gene–environment interactions (5, 12, 14, 16, 23, 24), building on computational toxicology approaches (28, 31).

## 5 Conclusion

This systematic review consolidates evidence suggesting a potential role for GBA disruption in EDC-induced PP, based largely on animal studies using experimental doses that often exceed human environmental exposure levels. Importantly, the conclusion that the GBA pathway may dominate at “low doses” is derived from rodent studies employing doses that are not representative of real-world human exposure. Therefore, the relevance of these findings to human populations exposed to environmentally relevant EDC mixtures (≤1 μg/kg/day) remains speculative and requires direct validation. Therefore, the relevance of these findings to human populations exposed to environmentally relevant EDC mixtures (≤1 μg/kg/day) remains speculative and requires direct validation through future research prioritizing models that employ human-relevant, low-dose mixtures. The evidence also points to significant synergistic interactions between EDCs in mixtures, although this conclusion is based on a very limited subset of studies (only 3.4% of those reviewed). This highlights a major challenge for current regulatory paradigms, which often do not fully address mixture toxicity and non-monotonic dose responses, partly due to a foundational lack of mixture toxicity data in mechanistic research.

These insights underscore the need for risk assessment strategies that integrate multi-omics biomarkers and prioritize evaluating complex, environmentally relevant mixtures. Future longitudinal human studies, investigations using humanized-microbiota animal models exposed to real-world EDC mixtures, and research into microbiota-targeted interventions are essential to validate these proposed mechanisms and inform effective public health policies aimed at reducing early-life EDC exposure. Addressing these challenges is crucial for developing evidence-based public health strategies to mitigate the risk of precocious puberty in an increasingly contaminated world.

Future longitudinal human studies, investigations using humanized-microbiota animal models exposed to real-world EDC mixtures, and research into microbiota-targeted interventions are essential to validate these proposed mechanisms and inform effective public health policies aimed at reducing early-life EDC exposure. Addressing these challenges is crucial for developing evidence-based public health strategies to mitigate the risk of precocious puberty in an increasingly contaminated world. Elucidating the role of the gut-brain axis in this process is not only a mechanistic advance but also a critical step towards protecting child developmental health from the insidious threat of environmental chemical mixtures.
