# Supplementary material for: The gut-brain axis mediates precocious puberty induced by environmentally relevant low-dose endocrine-disrupting chemical mixtures
Source: Front Endocrinol (Lausanne). 2026 Jan 22;16:1728811. doi: 10.3389/fendo.2025.1728811 (PMC12872493; doi:10.3389/fendo.2025.1728811)
Supplement: Supplementary file 1 [file DataSheet1.docx]

Supplementary Material

1.Supplementary Figure S1. PRISMA 2020 flow diagram of the study selection process.

The diagram outlines the phases of identification, screening, eligibility, and inclusion in the systematic review. A total of 4,582 records were identified through database searches, along with an additional 32 records obtained via manual searches (citation searching). After removing 1,485 duplicates, 3,129 unique records were screened based on their titles and abstracts, leading to the selection of 412 articles for full-text review. Of these, 325 articles were excluded for various reasons, with the most common being the failure to report on at least two sequential components of the hypothesized EDC–microbiota–GBA–PP pathway (n=255). In the end, 87 studies met all eligibility criteria and were included in the qualitative synthesis.

2.Supplementary Table S1: Complete Retrieval Formula

Structural requirements:

-Table by database (PubMed, Web of Science, etc.)

-Contains complete Boolean operators, truncation symbols, subject/free word combinations

-Annotate the search time range

#Search time range: January 1, 2010 to March 1, 2025

Final search update: 2025-05-01 (included El Kouche et al. 2025).

1) PubMed search equation

(

("Endocrine Disruptors"[Mesh] OR "Environmental Pollutants"[Mesh] OR

"Bisphenol A"[tw] OR "phthalate*"[tw] OR "DEHP"[tw] OR "PCB*"[tw])

AND

("Gastrointestinal Microbiome"[Mesh] OR "Dysbiosis"[Mesh] OR

"gut microbio*"[tw] OR "SCFAs"[tw] OR "bile acid*"[tw])

AND

("Puberty, Precocious"[Mesh] OR "precocious puber*"[tw] OR "GnRH"[tw])

)

AND (2010/01/01 : 2025/03/01[Date - Publication])

2) Web of Science search equation

TS=(

("Endocrine Disruptors" OR "Environmental Pollutants" OR "Bisphenol A" OR "phthalate" OR "DEHP" OR "PCB" OR "polybrominated diphenyl ethers" OR "PBDE" OR "perfluoroalkyl substances" OR "PFAS" OR "heavy metals" OR "lead" OR "cadmium" OR "chemical mixture" OR "EDC mixture" OR "pollutant cocktail")

AND

("Gastrointestinal Microbiome" OR "Dysbiosis" OR "gut microbio*" OR "microbiome" OR "Brain-Gut Axis" OR "vagus nerve" OR "SCFAs" OR "short chain fatty acid" OR "bile acid" OR "tryptophan" OR "inflammation" OR "intestinal barrier" OR "leaky gut" OR "tight junction")

AND

("Puberty, Precocious" OR "precocious puber" OR "early puber" OR "GnRH" OR "HPG axis" OR "kisspeptin")

)

Refined by: [Exclude meeting summary]

Timespan: 2010-2025

3) Scopus search equation

TITLE-ABS-KEY(

( "Endocrine Disruptors" OR "Environmental Pollutants" OR "Bisphenol A" OR phthalate OR DEHP OR PCB OR "polybrominated diphenyl ethers" OR PBDE OR "perfluoroalkyl substances" OR PFAS OR "heavy metals" OR lead OR cadmium OR "chemical mixture" OR "EDC mixture" OR "pollutant cocktail" )

AND

( "Gastrointestinal Microbiome" OR Dysbiosis OR "gut microbio" OR microbiome OR "Brain-Gut Axis" OR "vagus nerve" OR SCFAs OR "short chain fatty acid" OR "bile acid" OR tryptophan OR inflammation OR "intestinal barrier" OR "leaky gut" OR "tight junction" )

AND

( "Puberty, Precocious" OR "precocious puber" OR "early puber*" OR GnRH OR "HPG axis" OR "kisspeptin")

)

AND DOCTYPE( ar ) # Restricted Journal Articles

AND PUBYEAR > 2009 AND PUBYEAR < 2026

4) Embase search equation

(

('endocrine disruptor'/exp OR 'environmental pollutant'/exp OR 'bisphenol A' OR 'phthalate' OR 'DEHP' OR 'PCB' OR 'polybrominated diphenyl ether' OR 'PBDE' OR 'perfluoroalkyl compound'/exp OR 'PFAS' OR 'heavy metal'/exp OR 'lead'/exp OR 'cadmium'/exp OR 'chemical mixture' OR 'EDC mixture' OR 'pollutant cocktail')

AND

('gastrointestinal microbiome'/exp OR 'dysbiosis'/exp OR 'gut microbio' OR 'microbiome' OR 'brain gut axis'/exp OR 'vagus nerve'/exp OR 'SCFAs' OR 'short chain fatty acid' OR 'bile acid'/exp OR 'tryptophan'/exp OR 'inflammation'/exp OR 'intestinal barrier' OR 'leaky gut' OR 'tight junction')

AND

('precocious puberty'/exp OR 'precocious puber' OR 'early puber' OR 'GnRH'/exp OR 'HPG axis' OR 'kisspeptin'/exp)

)

AND [embase]/lim # Restricted Embase database

AND [2010-2025]/py # Publication year screening

3.Supplementary Table S2: PBPK Parameters for Dose Translation

| Parameter | Rat | Human | Source |
| --- | --- | --- | --- |
| Clearance (L/h/kg) | 0.32 | 0.11 | EPA/600/R-23/238 |
| Adipose:blood | 5.2 | 5.2 | McNally et al. 2021 |
